# Down-regulation of the inhibitor of growth family member 4 (ING4) in different forms of pulmonary fibrosis

**DOI:** 10.1186/1465-9921-10-14

**Published:** 2009-02-27

**Authors:** Argyris Tzouvelekis, Vassilis Aidinis, Vagelis Harokopos, Andreas Karameris, George Zacharis, Dimitrios Mikroulis, Fotios Konstantinou, Paschalis Steiropoulos, Ioannis Sotiriou, Marios Froudarakis, Ioannis Pneumatikos, Rodoula Tringidou, Demosthenes Bouros

**Affiliations:** 1 Department of Pneumonology, University Hospital of Alexandroupolis, Medical school, Democritus University of Thrace, Greece; 2 Institute of Immunology, Biomedical Sciences Research Center "Alexander Fleming", Athens, Greece; 3 Department of Pathology, VA Hospital (NIMTS), Athens, Greece; 4 Department of Cardiothoracic Surgery, University Hospital of Alexandroupolis, Medical school, Democritus University of Thrace, Greece; 5 Department of Intensive Care Medicine, University Hospital of Alexandroupolis, Medical school, Democritus University of Thrace, Greece; 6 Department of Pathology, General Hospital Sotiria, Athens, Greece

## Abstract

**Background:**

Recent evidence has underscored the role of hypoxia and angiogenesis in the pathogenesis of idiopathic fibrotic lung disease. Inhibitor of growth family member 4 (ING4) has recently attracted much attention as a tumor suppressor gene, due to its ability to inhibit cancer cell proliferation, migration and angiogenesis. The aim of our study was to investigate the role of ING4 in the pathogenesis of pulmonary fibrosis both in the bleomycin (BLM)-model and in two different types of human pulmonary fibrosis, including idiopathic pulmonary fibrosis (IPF) and cryptogenic organizing pneumonia (COP).

**Methods:**

Experimental model of pulmonary fibrosis was induced by a single tail vein injection of bleomycin in 6- to 8-wk-old C57BL/6mice. Tissue microarrays coupled with qRT-PCR and immunohistochemistry were applied in whole lung samples and paraffin-embedded tissue sections of 30 patients with IPF, 20 with COP and 20 control subjects.

**Results:**

A gradual decline of ING4 expression in both mRNA and protein levels was reported in the BLM-model. ING4 was also found down-regulated in IPF patients compared to COP and control subjects. Immunolocalization analyses revealed increased expression in areas of normal epithelium and in alveolar epithelium surrounding Masson bodies, in COP lung, whereas showed no expression within areas of active fibrosis within IPF and COP lung. In addition, ING4 expression levels were negatively correlated with pulmonary function parameters in IPF patients.

**Conclusion:**

Our data suggest a potential role for ING4 in lung fibrogenesis. ING4 down-regulation may facilitate aberrant vascular remodelling and fibroblast proliferation and migration leading to progressive disease.

## Introduction

Idiopathic interstitial pneumonias (IIPs) are a heterogeneous group of diffuse parenchymal diseases comprising of seven distinct clinical and pathological entities[1]. Among others idiopathic pulmonary fibrosis (IPF) and cryptogenic organizing pneumonia (COP) represent two of the most prevalent members of the disease group with major differences in pathogenesis, clinical course and prognosis. IPF is a refractory and lethal IIP characterized by fibroblast proliferation, extracellular matrix deposition and progressive lung scarring, comprising the histopathologic pattern of usual interstitial pneumonia (UIP)[2]. The incidence of IPF is estimated at 15–40 cases per 100,000 per year, and the mean survival from the time of diagnosis is 3–5 yr regardless of treatment [3]. Despite intense research efforts, their aetiopathogenesis is still elusive and controversial and consequently their treatment ineffective [4-6].

Inhibitor of growth family member 4 (ING4) languished in relative obscurity until the past three years when it emerged to function as a tumor suppressor gene, repressing cell proliferation[7], tumor growth[8], loss of contact inhibition [8-10] and angiogenesis[10]. ING4 belongs to a family of proteins comprising six members characterized by a highly conserved C-terminal plant homedomain (PHD)-like zinc-finger domain and has been implicated in a variety of processes including oncogenesis, apoptosis, DNA repair and cell cycle control[11]. Although, its precise mechanism of action has yet to be elucidated, ING4 seems to inhibit angiogenesis through interaction with hypoxia inducible factor (HIF) proly hydroxylases (HPH)[12,13] and RelA subunit of NF-Kβ [14]resulting in downregulation of *HIF *activation[12,13] and repression of angiogenesis related genes including *IL6, IL-8 and Cox2*[14], respectively. We have recently performed comparative expression profiling of disease progression in a well characterized animal model of pulmonary fibrosis and produced a number of highly involved genes in the disease pathogenesis. Among them, the role of HIF-1a signaling was further investigated and revealed overexpression of HIF-1a in the alveolar epithelium, both in the bleomycin-model and human pulmonary fibrosis suggesting a role in disease initiation and progression[15].

The aim of our study was to investigate the role of ING4 in the pathogenesis of pulmonary fibrosis by assessing its expression both in the bleomycin (BLM)-model and in two different types of human pulmonary fibrosis by using tissue microarrays, quantitative reverse transcription -(qRT)-polymerase chain reaction (PCR) and immunohistochemistry. ING4 was found downregulated in both mRNA and protein level within fibrotic lungs compared to controls whereas a gradual decline of ING4 expression following disease progression was noticed in the experimental model of pulmonary fibrosis. The expression pattern of ING4 within fibrotic lungs was inversely related with that of HIF-1a, as has previously been demonstrated, suggesting a role for this transcription factor during disease pathogenesis. Most intriguingly, ING4 semi-quantitative expression levels were negatively correlated with pulmonary function parameters in IPF patients, further supporting the premise that ING4 could potentially serve as a biomarker of disease progression.

## Materials and methods

### BLM-induced pulmonary inflammation and fibrosis

All mice strains were bred and maintained in the C57BL/6 background for over 20 generations in the animal facilities of the Biomedical Sciences Research Center "Alexander Fleming" under specific pathogen-free conditions, in compliance with the Declaration of Helsinki principles. Mice were housed at 20–22°C, 55 ± 5% humidity, and a 12 h light-dark cycle; food and water was given ad libitum. All experimentation was approved by an internal Institutional Review Board, as well as by the veterinary service and fishery department of the local governmental prefecture. Pulmonary Fibrosis was induced by a single tail vein injection of Bleomycin hydrogen chloride (100 mg/kg body weight; 1/3 LD50; Nippon Kayaku Co. Ltd., Tokyo) to 6- to 8-wk-old mice as previously reported in detail[16].

### Patients

In total, 50 newly diagnosed patients with IIPs of two different histopathologic patterns including 30 patients with IPF/UIP and 20 with COP were recruited in our study. The diagnosis was based on the consensus statement of the ATS/ERS (2002)[1]. Paraffin-embedded surgical lung specimens (open lung biopsy or by video assisted thoracoscopic surgery-VATS) from two different fibrotic regions of each individual were sampled. Approval by the local ethical committee was obtained. Twenty control paraffin blocks obtained from the normal part of lungs removed for benign lesions were collected from the archives of the Department of Pathology of three different institutions (Table 1).

### Quantitative Real-Time reverse transcriptase-polymerase chain reaction (qRT-PCR)

qRT-PCR was performed using the Chromo 4 Real-Time Detection System and the Platinum^® ^SYBR^® ^Green qPCR SuperMix-UDG (Invitrogen), according to the manufacturer's instructions. The program used included: 2 min at 50°C, 5 min at 95°C, 43 cycles of denaturation-annealing-extension (30s at 95°C; 45s at 56°C; 30s at 72°C) and a final extension of 5 min at 72°C. Primers were chosen from exons separated by large introns (spanning exon-exon junctions), and the PCR quality and specificity was verified by melting curve analysis and gel electrophoresis. Mouse (m) and human (h) primer sequences (s: sense, as: antisense) and expected lengths (in bp) were as follows (5' to 3'): (m) *Ing4 *(s: AAG GCC GGA CCC AAA AGG AG; as: CCA ACA CAT CAG AGG GGT GG; 171 bp), (h) *ING4 *(s: AGC TTG CCA TGC AGA CCT; as: GCG CAC GAG CTT TAA CTT; 245 bp). (m) *B2m *(s: TTC TGG TGC TTG TCT CAC TGA; as: CAG TAT GTT CGG CTT CCC ATTC; 104 bp). (h) *B2M *(s: CTG ACC CTA CAT TTT GTG CAT AAAAG ATG AGT ATG CC; as: ACC CTA CAT TTT GTG CAT AA; 202 bp). Cycle threshold (Ct; the first cycle that amplification can be detected) values were obtained from the Opticon monitor 3 software for each gene of interest and the control reference gene, together with amplification efficiencies (85–115%). Ct values were normalized to the reference gene beta-2-microglobulin (B2m/B2M for mouse and human respectively)[16].

For mouse samples the relative quantification method was used. This method determines the changes in steady-state mRNA levels of a gene of interest (GOI) across samples and expresses it relative to the levels of the control sample (23c in this case). The relative quantification Microsoft^® ^Excel add-on macro (*Bio-Rad *Laboratories) that utilizes the following mathematical model used to calculate relative expression of GOI.

$\text{Relative expression}=2^{-(\Delta {\text{Ct}}_{\text{Sample}}-\Delta {\text{Ct}}_{\text{control}})}$, where ΔCt = Ct_GOI _- Ct_Reference_. 5 mice per group/time point were utilized (d7, d15, d23); 3 in the control group (d23c). Equimolar amounts of the mouse cDNAs from each group were pooled together and were analyzed in triplicates. For the human samples, Ct values of both the GOI-*ING4 *and the reference gene B2M were converted to concentration values (ng/ml) utilizing a standard curve made by serial dilutions (in duplicates) of an arbitrary reference sample. *ING4 *concentration values were divided to the corresponding B2M values and presented as expression index.

### Tissue microarray (TMA) construction

A total of 70 tissue samples consisting of 30 IPF and 20 COP lung specimens and 20 control tissues derived from the normal part of lungs removed for benign lesions were snap-frozen and stored at -70°C. Specimens were fixed in cold-ethanol for 16 h and then embedded in paraffin. Hematoxylin and eosin (H&E) -stained sections were made from each block to define representative fibrotic and inflammatory lesion regions. Areas of interest were identified in H&E stained slides by a conventional microscope (Olympus BX-50). Tissue cylinders with a diameter of 1.5 mm were punched from selected areas of each "donor" block using a thin-wall stainless tube from a precision instrument (TMA-100, Chemicon, USA) and were transfered by a solid stainless stylet into defined array coordinates in a 45 * 20 mm new recipient paraffin block[17]. The tissue microarray blocks were constructed in three copies (each containing one sample from a different region of all lesions). One sample was taken from the center and two samples from different peripheral areas. Ultimately, we constructed two tissue microarray blocks comprising of 100 tissue elements each. Each tissue element in the array was 1.5 mm in diameter and spacing between two adjacent elements was 0.1 mm. After the tissue microarray construction 3 μm and 5 μm sections for immunohistochemical analysis, respectively, were cut from the "donor" blocks and were transferred to glass slides using an adhesive-coated tap sectioning system.

### Immunohistochemistry analysis

Immunohistochemistry for ING4 antigen was carried out on using the anti-h-ING4 rabbit polyclonal unconjugated antibody (10617-1-AP-Proteintech Group, Inc., Chicago, IL, USA) and the anti-ING4 mouse polyclonal antibody (Novus Biologicals Inc., Littleton, CO). The slides were deparaffinized and En Vision immunohistochemistry protocol (DAKO Corp, Denmark) was performed by the use of an automated immunohistochemistry staining system (Bond-Biogenex, USA). Diaminobenzidine (DAB) was used as chromogenic substrate. This immunohistochemistry protocol is based on a water-soluble, dextran polymer system preventing the endogenous biotin reaction, which is responsible for the background in the stained slides. More specifically, the sections were incubated with the primary antibody in "antibody diluent" (DAKO) and goat-anti-mouse EnVision- HRP-enzyme conjugate was performed for 3 min each. The "highly sensitive 3,3,' diaminobenzidine plus" (DAB+) and the "3-amino-9-ethylcarbazol plus" (AEC+) chromogens (both from DAKO) were used as substrates for the EnVision- HRP-enzymes. Staining intensity was further enhanced by modifying the manufacturer's protocol in that all incubation steps (primary antibodies, EnVision, and substrate reactions) were performed on slides placed horizontally on a thermal plate at 37C. After each incubation, the slides were dipped in TBS or, after the substrate reaction, in tapwater at RT and waved at maximum speed for 10 sec. Excess liquid (buffer/water) was soaked up by a paper towel. Specimens of colon adenocarcinoma cases were used as positive controls for the marker.

### Evaluation of results by Computerized Image Analysis

In order to evaluate the immunohistochemistry results not in a qualitative way but in a more accurate and reliable way, we performed computerized image analysis by using a semi-automated system (Matrox II Card Frame Grabber, Camera Microwave Systems, Microscope Olympus BX-50) allowing us to assess staining intensity in a 256 level scale – 0 (black)-255(white). Staining intensity values were then converted to reverse percentages {reverse staining intensity = (1-staining intensity/256) ×100}

### Statistical analysis

Statistical analysis was carried out using SPSS 14.0 software. Results are expressed as mean ± SD, or median (range), unless otherwise indicated. One way ANOVA was used to compare reverse staining intensity values between the three groups of subjects. In addition, statistical significance was further verified by performing independent samples t-test to compare reverse staining intensity values of ING4 between different forms of pulmonary fibrosis and between patients and controls. Results were corrected using Bonferroni correction. Spearman's correlation was used to find relationship between pulmonary function parameters and semi-quantitative expression levels of ING4, in IPF patients. A p-value of < 0.05 was considered as statistically significant.

## Results

### Decreased ING4 expression in the BLM model of pulmonary fibrosis following disease progression

**Figure 1:**
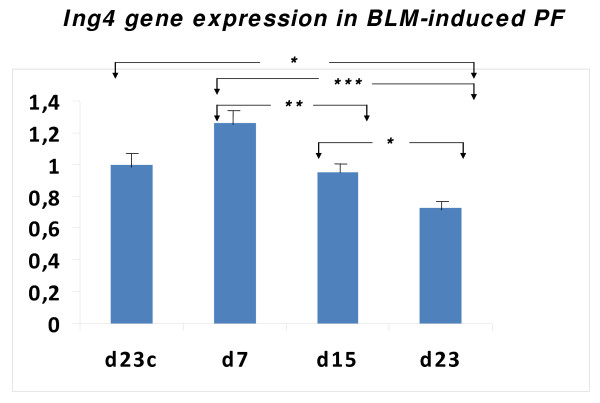
***Ing4 *mRNA expression levels in BLM-induced pulmonary fibrosis**. *Ing4 *gene expression levels quantified by qRT-PCR showed a trend to increase, compared to control untreated mice, at early disease stages (day 7 post-administration) whereas a gradual decline, compared to control and day 7 mice, following disease progression (days 7 and 15) was easily noted. All values were normalized with the reference gene *B2m *and presented as relative expression to the control sample as described in materials and methods. *p < 0.05, **p < 0.005, ***p < 0.001. (One way ANOVA).

**Figure 2:**
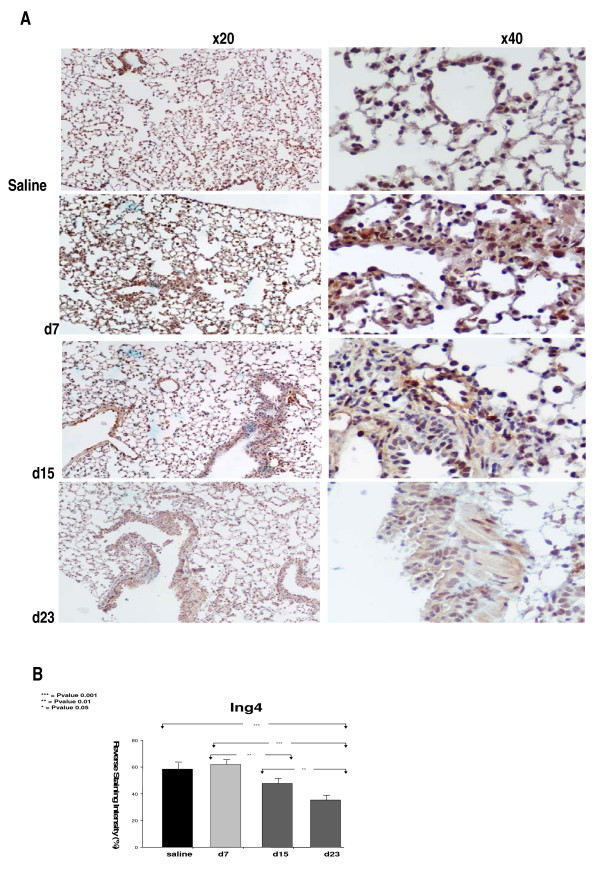
**Decreased ING4 expression in bleomycin (BLM)- induced pulmonary fibrosis (PF) following disease progression**. (A) Representative immunohistochemistry with an anti-ING4 antibody on lung paraffin sections from BLM-treated mice (7, 15, and 23, days post-administration). ING4 was mainly expressed in alveolar epithelium (days 7 and 15) whereas showed weak staining within areas of dense fibrosis and collagen deposition at late disease stages (day 21). (B) Computerized image analysis of immunostained sections. *p < 0.05, **p < 0.005, ***p < 0.001. (One way ANOVA and unpaired t-test with Bonferroni correction, F = 71,126).

As angiogenesis[15,18,19] and apoptosis[20,21] represent two of the major pathogenetic hallmarks of pulmonary fibrosis and since HIF-1a, the major transcription factor of hypoxia-related genes involved in angiogenesis and apoptosis, has been recently implicated in the pathogenesis of fibrotic lung disease we sought to investigate the expression of its inhibitor, ING4, both in mRNA and protein level using qRT-PCR and immunohistochemistry analysis, respectively, in a well characterized model of pulmonary fibrosis. Surprisingly, following disease progression, ING4 expression was found downregulated, both in mRNA and protein level, as shown in Figures 1 and 2. In particular, qRT-PCR analysis demonstrated that *Ing4 *gene expression was downregulated upon administration of BLM and the development of pulmonary inflammation and fibrosis (Figure 1). Experimental findings were further extended by immunohistochemistry analysis for ING4 expression on lung paraffin sections from BLM treated mice (7, 15 and 23 days post administration) which confirmed decreased expression during disease progression. ING4 was extensively expressed in normal epithelium in control lung samples, as well as in early stages of disease (day 7) where inflammation is prominent and fibrosis is almost absent (Figure 2).

### Decreased expression of ING4 within IPF lung compared to COP and control samples

**Figure 3:**
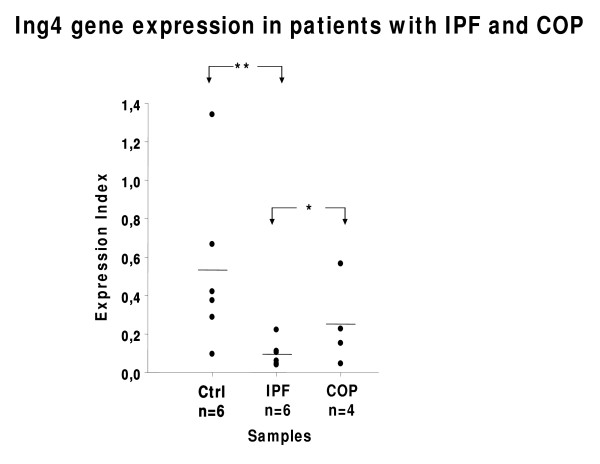
***ING4 *mRNA expression levels in patients with idiopathic pulmonary fibrosis (IPF), cryptogenic organizing pneumonia (COP) and control (ctrl) subjects**. Significant reduction of *ING4 *gene expression levels in IPF patients compared to COP and control subjects, as quantified by qRT-PCR. Cycle threshold (Ct) values for each sample were converted to concentration values (through a standard curve of serial dilutions of a reference sample), normalized to the corresponding values of the reference gene *B2M *and presented as expression index. *p < 0.05, **p < 0.005, ***p < 0.001 (One way ANOVA).

**Figure 4:**
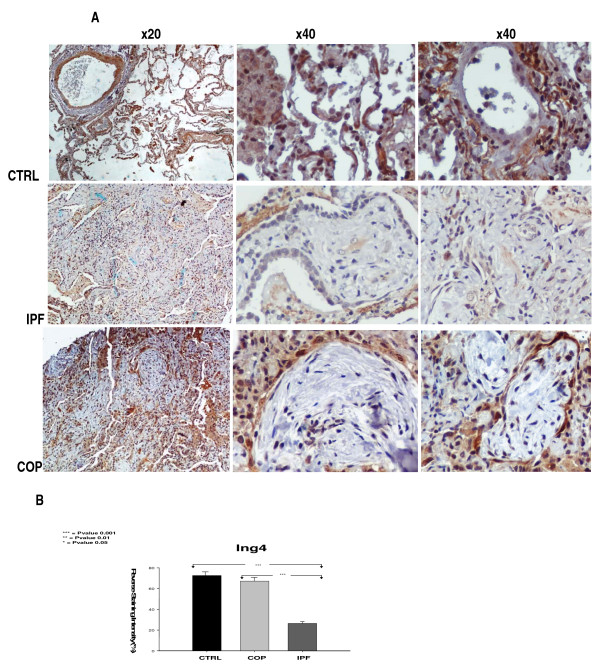
**Decreased ING4 expression within IPF lung compared to COP and normal lung**. (A) Representative immunohistochemistry with an anti-ING4 antibody on lung paraffin sections from IPF and COP patients as well as control (CTRL) subjects. ING4 was extensively expressed in normal alveolar epithelial and endothelial cells in control lung samples and was also visualized in alveolar epithelial cells surrounding areas of active fibrosis, called Masson bodies, within COP lung. On the contrary, ING4 was almost absent in alveolar epithelium and fibrotic interstitium (fibroblastic foci) within IPF lung. (B) Computerized image analysis of immunostained sections. *p < 0.05, **p < 0.005, ***p < 0.001 (One way ANOVA and unpaired t-test with Bonferroni correction, F = 171,126).

Because BLM-model of pulmonary fibrosis is not fully representative of IPF due to its self limiting nature, rapidity of its development and close association between lung injury and inflammation[22], we sought to extend our observations in patients with IPF and COP, two different types of pulmonary fibrosis with different disease progressiveness and treatment responsiveness. In accordance with results showed in our experimental model of pulmonary fibrosis, *Ing4 *gene expression was downregulated in four available IPF compared to six controls and four COP whole lung samples (Figure 3). The samples included in this analysis were representative of a total number of 70 tissue samples (30 IPF, 20 controls and 20 COP) used for TMA construction and immunohistochemistry semi-quantitative analysis which further corroborated ING4 down-regulation in IPF patients compared to controls and COP subjects, on a protein level as well (Figure 4). In particular, ING4 showed strong staining intensity within normal epithelium and endothelium, in almost 90% of control lung samples whereas it was also visualized in alveolar epithelial cells surrounding areas of active fibrosis, also known as Masson bodies, within the COP lung (80% or 16/20 patients). No statistical difference in staining intensity was observed between COP and control lung samples in ING4 expression (Figure 4). On the contrary, ING4 was almost absent within IPF lung in the majority of IPF patients (80%), including areas of active fibrosis, also called fibroblastic foci, as well as alveolar epithelial cells immediately adjacent to them (Figure 4).

### ING4 semi-quantitative expression levels were negatively correlated with pulmonary function parameters in IPF patients

**Figure 5:**
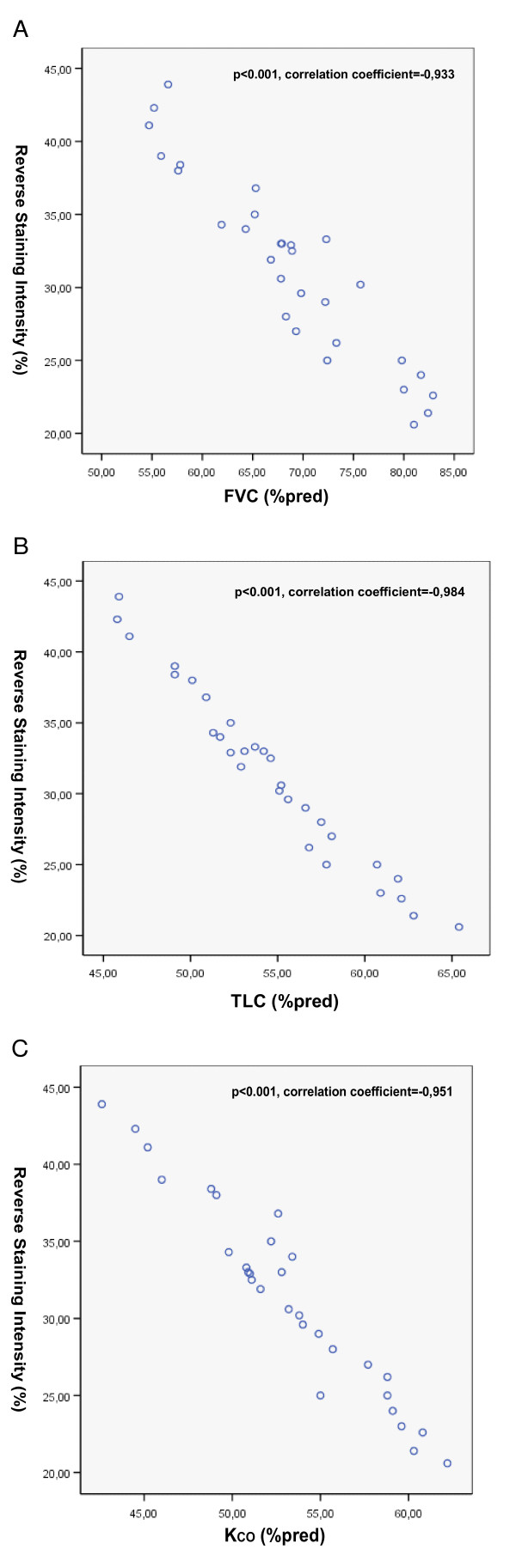
**Negative correlation between ING4 semi-quantitative expression levels and pulmonary function parameters in IPF patients**. Spearman's correlation was performed and clearly demonstrated an almost linear negative association between ING4 down-regulation and parameters of disease progression including including forced vital capacity (FVC) (A), total lung capacity (TLC) (B) and diffuse lung capacity as expressed by K_CO _(carbon monoxide transfer coefficient) (C), in IPF patients.

To strengthen the evidence that decreased ING4 expression may contribute to the progression of fibrosis we sought to correlate ING4 semi-quantitative immunohistochemistry expression levels with pulmonary function parameters including forced vital capacity (FVC), total lung capacity (TLC) and diffuse lung capacity as expressed by K_CO _(carbon monoxide transfer coefficient), in IPF patients. Most intriguingly statistical analysis clearly demonstrated an almost linear negative relationship between ING4 down-regulation and FVC (p < 0.001, correlation coefficient = -0,933), TLC (p < 0.001, correlation coefficient = -0,984) and finally K_CO _(p < 0.001, correlation coefficient = -0,951), as shown in Figure 5(A, B) and 5(C) respectively.

## Discussion

In the present study we analyzed, for the first time in the literature, the expression profiles of ING4 in the experimental model of pulmonary fibrosis as well as in patients with two different forms of fibrotic lung disease, IPF and COP. ING4 is a candidate tumor suppressor gene that functions in cell proliferation, contact inhibition and angiogenesis. Seminal observations have attributed ING4 a beneficial role in regulating cancer invasion, migration and metastasis and highlighted it as a novel therapeutic target[23]. ING4 is widely expressed in normal cell lines while it is down-regulated in glioblastoma[24] and melanoma cells[23] as well as in head and neck squamous cell carcinoma[25]. ING4 negatively regulates cell proliferation leading to growth inhibition through its capability to interact with p300 and consequently enhance p53 acetylation, promoting p53-dependent apoptosis[8].

The last fifteen years parallels have been drawn between lung cancer and pulmonary fibrosis. The unremitting recruitment and maintenance of the altered fibroblast phenotype with generation of highly-proliferative immortal fibroblasts[22,26,27] coupled with the epithelial-mesenchymal transition (EMT)[22,28-32] phenomenon is reminiscent with the transformation of cancer cells and metaplasia. Additionally, recently emerged evidence implicating increased angiogenic activity and aberrant vascular remodeling in the etiopathogenesis of pulmonary fibrosis has attracted much attention[15,18,19,28,33,34]. However, despite intense research efforts the pathogenetic cascade of fibrotic lung disease still remains elusive and controversial. To this end and in an attempt to scrutinize for novel disease mediators, our study group recently identified HIF-1a and other hypoxia related genes as the most deregulated during disease progression in a well characterized animal model of pulmonary fibrosis. Extending beyond target identification, the role of HIF-1a signaling was further explored with a series of experiments which revealed overexpression in the hyperplastic epithelium of IPF patients, colocalizing with its target genes, p53 and vascular endothelial growth factor (VEGF), involved in apoptosis and angiogenesis, respectively[15].

Following the above series of experiments and to shed further light on the fibrogenic cascade, we sought to determine the expression profiles of ING4, also known as inhibitor of HIF-1a, in different forms of pulmonary fibrosis, including the experimental model and two types of idiopathic fibrotic lung disease, IPF and COP. The latters, while belonging at the same disease group they present with different clinical course and treatment responsiveness that may be attributed to distinct apoptotic and angiogenic profiles. Interestingly, we noticed a significant down-regulation of ING4 expression, both in mRNA and protein level, in the BLM-model of pulmonary fibrosis compared to untreated mice. Intriguingly, reduced expression of ING4 was noted as a gradual decline in parallel with disease progression. As shown, in figures 1 and 2, ING4 was widely expressed at early stages of the disease (day 7) (a trend toward increased expression was noticed at this time point compared to control untreated mice), whereas showed significant reduction as disease was progressing from mild inflammation towards dense fibrosis and areas of architectural distortion (day 21). This expression pattern is opposing to that seen for HIF-1a, where a gradual increase following disease progression was demonstrated. On the basis of the anti-proliferative, anti-angiogenic and anti-oncogenic properties of ING4, one can easily suggest that the above expression pattern (trend to increase as disease starts to develop and significant decline as disease progresses) may represent a protective compensatory response of the epithelium against the injurious stimulus of BLM-administration.

Because BLM-model of pulmonary fibrosis is not fully representative of IPF we sought to enhance our findings in patients with IPF and COP. In line with animal results, both mRNA and protein levels of ING4 were found down-regulated in IPF compared to COP patients and normal subjects. Our data are in accordance with findings in cancer cell lines and patients who exhibited dramatically decreased ING4 levels which correlated with poorer survival and low treatment responsiveness. Most intriguingly, ING4 was almost absent within IPF lung while showed only prominent staining in the alveolar epithelium surrounding areas of active fibrosis, called Masson bodies, within COP lung. Although speculative, it is reasonable to assume that ING4 is reduced in more progressive and irreversible forms of pulmonary fibrosis and its suppression may abrogate its versatile protective properties contributing to rapid disease progression and poor treatment responsiveness.

ING4 down-regulation may be explained both by genetic and environmental factors. In particular, it has been proposed that the reduction of ING4 expression in head and neck squamous cell carcinoma as well as in glioblastoma[24] and melanoma[23,35] patients maybe attributed to mutations or deletions at chromosome 12p12-13, which includes *Ing4 *gene. Whether patients with sporadic or familial IPF present with mutations in *Ing4*, as it happens with telomerase [36-39], and whether these mutations affect ING4 expression and are associated with patients' survival and treatment response, remains to be proven.

Finally, in an attempt to support our premise that ING4 downregulation may contribute to lung fibrosis and lead to more progressive disease stages, we have demonstrated that ING4 semi-quantitative expression levels are negatively associated with markers of disease prognosis including pulmonary function parameters such as FVC, TLC and K_CO_, as shown in Figure 5(A, B) and 5(C) respectively. In addition, this linear correlation may indicate ING4 as a potential biomarker that could reliably predict clinical course and treatment response in IPF patients. However, future longitudinal studies in a large number of well defined patients are sorely needed to support this provocative hypothesis.

Despite relative enthusiasm arising from the above observations, our data exhibit a number of limitations that should be treated with caution. Firstly, based on our findings, it is rather unclear whether ING4 down-regulation within fibrotic lung is a primary event or just a consequence of the fibrogenic cascade. However, in order for a causal-effect relationship to be proven, generation of ING4 knockout and/or transgenic mice is sorely needed. Secondly, based on our approach it is not definitive whether ING4 inactivation leads to abrogation of these protective (anti-migratory, anti-angiogenic) properties and is partially responsible for poor patients' survival. Nevertheless, our study represents the first attempt to implicate a novel tumor-suppressor protein in the pathogenesis of pulmonary fibrosis and to associate its relatively obvious absence with disease development and progression.

Collectively our dataset demonstrates for the first time in the literature down-regulation of ING4 in different forms of pulmonary fibrosis. Reduced expression of ING4 may facilitate aberrant vascular remodelling and fibroblast proliferation and migration leading to progressive disease and culminating to a fatal outcome. Our observations suggest that ING4 may serve as a reliable prognosticator as well as a potential therapeutic target for a group of diseases with unfavourable prognosis and yet ineffective treatment. Future prospective studies in patients with different types of fibrotic lung disease searching for *Ing4 *mutations coupled with experimental data using ING4 knockout mice may provide a way forward.

## Competing interests

The authors declare that they have no competing interests.

## Authors' contributions

AT, VA and DB were involved with the study conception. AT, PS, IS, MF, GZ, MK, FK and DB recruited the patients in the study. AT and MF performed the statistical analysis of the manuscript. AT carried out the semi-quantitative immunohistochemical computerized image analysis of the tissue sections. AT and AK constructed the tissue microarrays. RT set the histological diagnosis of IIPs and provided us with the controls tissue samples. VH performed the qRT-PCR and the bleomycin-induced PF model. AT prepared the manuscript. DB, VA, MF and IP were involved in revising the article for important intellectual content. All authors read and approved the final manuscript.

## References

[B1] (2002). American Thoracic Society/European Respiratory Society International Multidisciplinary Consensus Classification of the Idiopathic Interstitial Pneumonias. This joint statement of the American Thoracic Society (ATS), and the European Respiratory Society (ERS) was adopted by the ATS board of directors, June 2001 and by the ERS Executive Committee, June 2001. Am J Respir Crit Care Med.

[B2] American Thoracic Society (2000). Idiopathic pulmonary fibrosis: diagnosis and treatment. International consensus statement. American Thoracic Society (ATS), and the European Respiratory Society (ERS). Am J Respir Crit Care Med.

[B3] Raghu G, Weycker D, Edelsberg J, Bradford WZ, Oster G (2006). Incidence and prevalence of idiopathic pulmonary fibrosis. Am J Respir Crit Care Med.

[B4] Selman M, Pardo A, Kaminski N (2008). Idiopathic pulmonary fibrosis: aberrant recapitulation of developmental programs?. PLoS Med.

[B5] Bouros D, Antoniou KM (2005). Current and future therapeutic approaches in idiopathic pulmonary fibrosis. Eur Respir J.

[B6] Selman M, King TE, Pardo A (2001). Idiopathic pulmonary fibrosis: prevailing and evolving hypotheses about its pathogenesis and implications for therapy. Ann Intern Med.

[B7] Shen JC, Unoki M, Ythier D (2007). Inhibitor of growth 4 suppresses cell spreading and cell migration by interacting with a novel binding partner, liprin alpha1. Cancer Res.

[B8] Garkavtsev I, Kozin SV, Chernova O (2004). The candidate tumour suppressor protein ING4 regulates brain tumour growth and angiogenesis. Nature.

[B9] Kim S, Chin K, Gray JW, Bishop JM (2004). A screen for genes that suppress loss of contact inhibition: identification of ING4 as a candidate tumor suppressor gene in human cancer. Proc Natl Acad Sci USA.

[B10] Kim S (2005). HuntING4 new tumor suppressors. Cell Cycle.

[B11] Campos EI, Chin MY, Kuo WH, Li G (2004). Biological functions of the ING family tumor suppressors. Cell Mol Life Sci.

[B12] Ozer A, Bruick RK (2005). Regulation of HIF by prolyl hydroxylases: recruitment of the candidate tumor suppressor protein ING4. Cell Cycle.

[B13] Ozer A, Wu LC, Bruick RK (2005). The candidate tumor suppressor ING4 represses activation of the hypoxia inducible factor (HIF). Proc Natl Acad Sci USA.

[B14] Colla S, Tagliaferri S, Morandi F (2007). The new tumor-suppressor gene inhibitor of growth family member 4 (ING4) regulates the production of proangiogenic molecules by myeloma cells and suppresses hypoxia-inducible factor-1 alpha (HIF-1alpha) activity: involvement in myeloma-induced angiogenesis. Blood.

[B15] Tzouvelekis A, Harokopos V, Paparountas T (2007). Comparative expression profiling in pulmonary fibrosis suggests a role of hypoxia-inducible factor-1alpha in disease pathogenesis. Am J Respir Crit Care Med.

[B16] Oikonomou N, Harokopos V, Zalevsky J (2006). Soluble TNF mediates the transition from pulmonary inflammation to fibrosis. PLoS ONE.

[B17] Kononen J, Bubendorf L, Kallioniemi A (1998). Tissue microarrays for high-throughput molecular profiling of tumor specimens. Nat Med.

[B18] Antoniou KM, Tzouvelekis A, Alexandrakis MG (2006). Different angiogenic activity in pulmonary sarcoidosis and idiopathic pulmonary fibrosis. Chest.

[B19] Tzouvelekis A, Anevlavis S, Bouros D (2006). Angiogenesis in interstitial lung diseases: a pathogenetic hallmark or a bystander?. Respir Res.

[B20] Plataki M, Koutsopoulos AV, Darivianaki K, Delides G, Siafakas NM, Bouros D (2005). Expression of apoptotic and antiapoptotic markers in epithelial cells in idiopathic pulmonary fibrosis. Chest.

[B21] Uhal BD (2003). Epithelial apoptosis in the initiation of lung fibrosis. Eur Respir J Suppl.

[B22] Phan SH (2003). Fibroblast phenotypes in pulmonary fibrosis. Am J Respir Cell Mol Biol.

[B23] Li J, Martinka M, Li G (2008). Role of ING4 in human melanoma cell migration, invasion and patient survival. Carcinogenesis.

[B24] Hassler M, Seidl S, Fazeny-Doerner B (2006). Diversity of cytogenetic and pathohistologic profiles in glioblastoma. Cancer Genet Cytogenet.

[B25] Gunduz M, Nagatsuka H, Demircan K (2005). Frequent deletion and down-regulation of ING4, a candidate tumor suppressor gene at 12p13, in head and neck squamous cell carcinomas. Gene.

[B26] Liu T, Nozaki Y, Phan SH (2002). Regulation of telomerase activity in rat lung fibroblasts. Am J Respir Cell Mol Biol.

[B27] Liu T, Chung MJ, Ullenbruch M (2007). Telomerase activity is required for bleomycin-induced pulmonary fibrosis in mice. J Clin Invest.

[B28] du Bois RM (2007). Mechanisms of scleroderma-induced lung disease. Proc Am Thorac Soc.

[B29] Kasai H, Allen JT, Mason RM, Kamimura T, Zhang Z (2005). TGF-beta1 induces human alveolar epithelial to mesenchymal cell transition (EMT). Respir Res.

[B30] Willis BC, Liebler JM, Luby-Phelps K (2005). Induction of epithelial-mesenchymal transition in alveolar epithelial cells by transforming growth factor-beta1: potential role in idiopathic pulmonary fibrosis. Am J Pathol.

[B31] Willis BC, duBois RM, Borok Z (2006). Epithelial origin of myofibroblasts during fibrosis in the lung. Proc Am Thorac Soc.

[B32] Willis BC, Borok Z (2007). TGF-beta-induced EMT: mechanisms and implications for fibrotic lung disease. Am J Physiol Lung Cell Mol Physiol.

[B33] Strieter RM (2005). Pathogenesis and natural history of usual interstitial pneumonia: the whole story or the last chapter of a long novel. Chest.

[B34] Zisman DA, Keane MP, Belperio JA, Strieter RM, Lynch JP (2005). Pulmonary fibrosis. Methods Mol Med.

[B35] Unoki M, Shen JC, Zheng ZM, Harris CC (2006). Novel splice variants of ING4 and their possible roles in the regulation of cell growth and motility. J Biol Chem.

[B36] Armanios M, Chen JL, Chang YP (2005). Haploinsufficiency of telomerase reverse transcriptase leads to anticipation in autosomal dominant dyskeratosis congenita. Proc Natl Acad Sci USA.

[B37] Armanios MY, Chen JJ, Cogan JD (2007). Telomerase mutations in families with idiopathic pulmonary fibrosis. N Engl J Med.

[B38] Tsakiri KD, Cronkhite JT, Kuan PJ (2007). Adult-onset pulmonary fibrosis caused by mutations in telomerase. Proc Natl Acad Sci USA.

[B39] Alder JK, Chen JJ, Lancaster L (2008). Short telomeres are a risk factor for idiopathic pulmonary fibrosis. Proc Natl Acad Sci USA.

